# Comparative Pathogenicity of Duck Hepatitis A Virus-1 Isolates in Experimentally Infected Pekin and Muscovy Ducklings

**DOI:** 10.3389/fvets.2020.00234

**Published:** 2020-05-15

**Authors:** Islam Hisham, Hany F. Ellakany, Abdullah A. Selim, Mohammed A. M. Abdalla, Mohamed A. Zain El-Abideen, Walid H. Kilany, Ahmed Ali, Ahmed R. Elbestawy

**Affiliations:** ^1^Reference Laboratory for Veterinary Quality Control on Poultry Production (RLQP), Animal Health Research Institute, Damanhour, Egypt; ^2^Poultry and Fish Diseases Department, Faculty of Veterinary Medicine, Damanhour University, Damanhour, Egypt; ^3^Reference Laboratory for Veterinary Quality Control on Poultry Production (RLQP), Animal Health Research Institute, Giza, Egypt; ^4^Poultry Diseases Department, Faculty of Veterinary Medicine, Beni-Suef University, Beni Suef, Egypt

**Keywords:** duck hepatitis A virus-1 (DHAV-1), pathogenicity, pekin, muscovy, duckling, Egypt

## Abstract

Duck hepatitis virus (DHV) has always been considered one of the threats endangering duck farming in Egypt since the 1960s. In the current study, suspected DHV samples (*n* = 30) were obtained from commercial Pekin, Mulard (hybrid), and Muscovy duck farms and backyards in Beheira, Alexandria, Gharbia, Kafr El-Sheikh, and Giza provinces between 2012 and 2017. Diseased 3–21-day-old ducklings showed a clinical history of high mortality rates and nervous signs. Samples were screened by RT-PCR targeting the 5′UTR region and VP1 gene. The PCR-confirmed samples (*n* = 7) were isolated *via* allantoic route inoculation onto 9-day-old specific-pathogen-free embryonated chicken eggs. Embryos showed stunting, subcutaneous hemorrhages, and liver necrotic greenish-yellow foci. Duck hepatitis A virus-1 (DHAV-1) isolates were genetically analyzed in comparison to other field and vaccine strains. Phylogenetic analyses of the full-length VP1 gene sequences revealed that the obtained DHAV-1 field isolates clustered into genetic group 4 alongside other Egyptian strains isolated during the same period (95.9–99.72% similarity). Amino acid substitutions in the carboxyl-terminal of VP1 (I180T, G184E, D193N, and M213I) were identified in two strains. Also, deletion mutation at I189 was detected in three DHAV-1 strains. Additionally, the two amino acid residues E205 and N235 were common among the isolated strains and other virulent DHAV-1 strains. Two DHAV-1 isolates originated from Pekin source were selected for conducting the comparative pathogenicity testing based on detected point mutations at C-terminus of VP1. We evaluated the pathogenicity of these isolates by investigating clinical signs, mortality rates, and gross pathological and microscopic lesions. The study revealed that experimentally infected Pekin and Muscovy ducklings showed similar clinical signs including squatting down, lateral recumbency, and spasmodic kicking. Muscovy showed milder pathological changes in the liver compared to Pekin ducklings. Histopathological findings supported the gross pathological lesions detected in both breeds. In conclusion, these data provide updated information on the genetic diversity and pathotyping of Egyptian DHAV-1 strains. To the best of our knowledge, this is the first report of comparative pathogenicity of recent DHAV-1 strains in Pekin and Muscovy ducklings in Egypt and the Middle East region.

## Introduction

Duck viral hepatitis is an acute, highly contagious viral infection of young ducklings, characterized mainly by rapid onset and high mortality rates (50–95%). Duck hepatitis virus (DHV) was first reported in the United States in 1949 and was isolated in chicken embryos in 1950. Duck hepatitis A virus-1 (DHAV-1), classified as a sole member of Picornavirus genus (*Avihepatovirus*) (1), is the most virulent virus type which can cause mortality up to 95% in ducklings younger than 3 weeks old (2). DHAV-2, isolated in Taiwan (3), and DHAV-3, isolated in South Korea (4), Vietnam, (5), and China (6), are two newly described DHV genotypes, belonging to the same genus (*Avihepatovirus)* and can cause high mortalities in ducklings. Duck astrovirus type 1 (DAstV-1) and type 2 (DAstV-2 belonging to the genus (*Avastrovirus)* in the family *Astroviridae* in addition to a member of the hepadnavirus group [duck hepatitis B virus (DHBV)] are etiologically associated with liver disease in ducks as well (2).

The DHA is a positive-sense, single-stranded RNA virus that comprises ~7,623–7,691 nucleotides and contains a single open reading frame (ORF) encoding a polyprotein of 2,249 amino acids (7, 8). The polyprotein is processed into the capsid viral proteins VP0, VP3, and VP1. VP1 is the most external and immunodominant structural protein of the picornavirus capsid proteins since it contains motifs that interact with cellular receptors to elicit neutralizing antibodies (9).

In Egypt, the first record of DHV was reported in the late 1960s (10). After then, several studies have discussed the prevalence and epidemiological situation of DHV in Egypt (11–15). Recently, genetic analysis and molecular characterization of Egyptian DHAV-1 isolates have been described based on VP1 gene sequencing (16). The analyses showed that Egyptian DHAV-1 isolates have been clustered into genetic group 4 with virulent Chinese strains (17). Lately, the Egyptian DHAV-1 isolates were classified into three subgroups (A, B, and C) under genetic group 4 based on their geographical distribution (18).

DHAV-1 infection had only been associated with acute disease in Mallard and Pekin ducklings (19). However, it was reported that DHAV-1 may cause pancreatitis and encephalitis in Muscovy ducks (20, 21). Fu et al. (22, 23) demonstrated that pancreatitis in Muscovy ducklings was due to infection with a distinct pathotype of DHAV-1. These findings were supported when Muscovy ducklings exhibited pancreatic yellow discoloration or hemorrhage with a mortality rate of 25–40% in recent outbreaks of DHAV-1 in several regions of China (24).

In this study, we investigated the prevalence of DHAV-1 in Pekin, Mulard (hybrid), and Muscovy duck flocks exhibiting mortalities and hepatitis at some provinces in Egypt. DHAV-1 was screened, and virus isolation was attempted on specific-pathogen-free embryonated chicken eggs (SPF-ECEs). The isolated viruses were molecularly characterized based on the full-length VP1 gene sequencing. Additionally, the *in vivo* pathogenicity was tested in Pekin and Muscovy ducklings in terms of clinical signs, mortality, gross lesions, and histopathological changes.

## Materials and Methods

### Prevalence of Duck Hepatitis A Virus-1 in Duck Farms in Egypt

#### Case History and Gross Pathology

**Figure 1:**
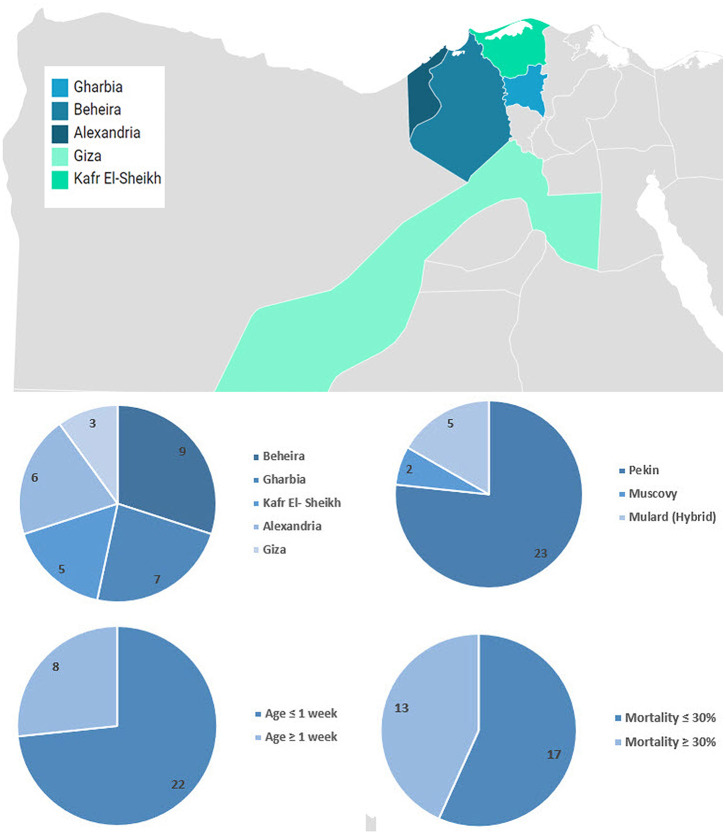
Geographical distribution and summary of collected samples. Map was created using Datawrapper free tool https://www.datawrapper.de/.

Liver samples (*n* = 30) were collected from 3 to 21-day-old commercial flocks and backyard ducklings (500–5,000 birds) between 2012 and 2017. Duck breeds included Pekin (*n* = 23), Mulard (*n* = 5), and Muscovy (*n* = 2). Diseased ducklings had a history of nervous signs and high mortalities (Supplementary Table 1). The distribution over the study period and the geographical location are shown in Figure 1.

#### RT-PCR Detection

Viral RNA was extracted from 45-μm filtered supernatants of liver tissue homogenates using the QIAamp Viral RNA Mini kit (Qiagen, Germany) according to the manufacturer's recommendations. The RT-PCR assays using oligonucleotide primers (Metabion, Germany) for partial amplification of 5′UTR (F: 5′-CCTCAGGAACTAGTCTGGA-3′, R: 5′-GGAGGTGGTGCTGAAA-3′) (6) and VP1 of DHAV-1 (F: 5′-ATCAGGGTGATTCTAACCAG-3′, reverse: 5′-CTTATTTCTAATTTGGTCAG-3′) (25). A 25-μl reaction containing 12.5 μl of Quantitect probe RT-PCR buffer (Qiagen, Germany, GmbH), 0.25 μl of RT-enzyme, 1 μl of each primer of 20 pmol concentration, 4.25 μl of water, and 6 μl of template RNA was used. The reactions were performed in a T3 thermal cycler (Biometra, Ireland). The amplicons were separated by electrophoresis on 1.5% agarose gel along with 100-bp DNA Ladder (Qiagen, Germany).

### Characterization of Recent Egyptian Duck Hepatitis A Virus-1 Viruses

#### Virus Isolation

Livers of PCR-confirmed samples were homogenized in sterile phosphate-buffered saline (PBS; pH 7.2) using T25 digital ULTRA-TURRAX® IKA (Germany) automatic homogenizer to form a 20% suspension (w/v) then subjected to three successive freeze–thaw cycles, and the supernatants were centrifugated at 500 g for 10 min then filtered by 0.2-μm syringe filters (Sartorius, USA). The supernatants of the liver homogenates were inoculated in the allantoic cavities of 9-day-old SPF-ECE (Nile SPF eggs, Koom Oshiem, Fayoum, Egypt) and incubated at 37°C. The inoculated (ECE) were candled daily for 7 days post-inoculation. Embryos were examined for stunting, subcutaneous hemorrhages, and pathological changes (liver, kidneys, and spleen). Allantoic fluid stocks were filtered by 0.2-μm syringe filters (Sartorius, USA) and then stored at −80°C until use.

#### Sequencing and Phylogenetic Analyses

The full VP1 gene of seven RT-PCR-positive field isolates from five Egyptian provinces was sequenced in both directions (Macrogen Inc., Korea). All sequences were aligned with DHAV-1 sequences retrieved from the GenBank database. Evolutionary analyses were conducted in MEGA X software. The evolutionary history was inferred using the Neighbor-Joining method with 1,000 bootstrap replicates. The evolutionary distances were computed using the maximum composite likelihood method, and the analysis involved 65 nucleotide sequences with a total of 749 positions in the final dataset. All ambiguous positions were removed for each sequence pair (pairwise deletion) (26). Nucleotide and amino acid alignment and identity percent were generated using Geneious® basic software 7.1.3 (Copyright © 2005–2014 Biomatters Ltd.). Sequences generated in this study were submitted to the GenBank.

### Experimental Infection of Duck Hepatitis A Virus-1 in Pekin and Muscovy Duckling

#### Ethical Approval

Animals were raised in compliance with the animal welfare regulations and maintained according to standard protocols. All experimental procedures were reviewed and approved by the Animal Care and Use Committee of the Faculty of Veterinary Medicine, Damanhour University, Egypt (Approval no. DMU/VetMed-2018/0213).

#### Experimental Ducklings

A total of 30 Pekin and 30 Muscovy ducklings of 1-day-old were purchased from a local hatchery from unvaccinated backyard breeders. Experimental ducklings were reared under strict hygienic measures inside negative-pressure isolators. Liver samples collected from additional three ducklings from each breed and tested for preexisting DHAV-1 infection using RT-PCR. Selected ducklings were subdivided into six groups (10 birds/group). Pekin groups (No. 1 and 2) were inoculated intramuscularly with DHAV-1 MK510860 (Eg/HL-1/15) and MK510859 (Eg/219/14) strains (10^5.0^ EID_50_ /0.5 ml), respectively. Similarly, Muscovy groups (No. 4 and 5) were inoculated with DHAV-1 strains in the same manner. The control groups of both Pekin and Muscovy ducklings (No. 3 and 6, respectively) were inoculated intramuscularly with 0.5 ml sterile PBS. Feed and water were available *ad libitum* (Table 1).

**Table 1:** Experimental design of duck hepatitis A virus-1 (DHAV-1) pathogenicity study in Pekin and Muscovy ducklings.

**Group**	**Experimental ducklings**		**Challenge virus**		
	**No**.	**Breed**	**Isolate**	**Dose (EID_**50**_/0.5 ml)**	**Route**
1	10	Pekin	MK510860 (Eg/HL1/15)	10^5.0^	Intramuscular
2	10		MK510859 (Eg/219/14)		
3	10		Non-challenged	PBS	—
4	10	Muscovy	MK510860 (Eg/HL1/15)	10^5.0^	Intramuscular
**5**	10		MK510859 (Eg/219/14)		
**6**	10		Non-challenged	PBS	—

#### Clinical Observation and Sample Collection

Ducklings were monitored daily for 7 days or until death. All clinical signs such as lethargy, nervousness with kicking, squatting, half-closed eyes, and death with opisthotonos were observed and recorded. The dead (or showing clinical signs) ducks were necropsied, and the gross lesions were recorded. Representative tissue samples from the liver, brain, and pancreas were collected from each group for histopathological examination.

#### Histopathological Examination

The tissues were fixed in a buffered formalin solution, then processed, embedded, and sectioned. The sections were stained with hematoxylin and eosin using standard procedures (27). Ordinal semiquantitative histopathologic scoring was developed for microscopic evaluation of detected tissue damage indices, i.e., degenerative changes, inflammation, and cell injury (–: normal; +: mild lesion; ++: moderate; + + +: severe; + + ++: very severe) (28).

## Results

### Prevalence of Duck Hepatitis A Virus-1 in Field Samples

Freshly dead ducklings from the affected farms showed drawn-back heads (opisthotonos position). The most consistent finding in the dead ducklings was enlarged liver with distinct punctate and ecchymotic hemorrhages. Mortality rates in diseased farms ranged between 5 and 80%. The average mortality in Pekin, Mulard (hybrid), and Muscovy ducklings were 36, 32.4, and 20%, respectively (Supplementary Table 1).

Primarily, 10 out of 30 suspected samples (33%) were positive for DHAV based on 5′ UTR RT-PCR (Pekin = 6/23, Mulard = 3/5, and Muscovy = 1/2). Beheira province contributed the largest portion of positive DHAV-1 samples with 40%, followed by Alexandria and Kafr El Sheikh with 20%, and Gharbia and Giza with 10% for each. Out of these 10 samples, seven samples were further confirmed through the amplification of the genomic region of VP1 of DHAV-1 (Supplementary Table 1). The positivity of samples originated from Pekin ducklings was 6 out of 6 (100%), Mulard was 1 out of 3 (33.3%), while the Muscovy 5′UTR RT-PCR positive sample was negative for VP1 of DHAV-1. All confirmed DHAV-1 samples proved to be free from extraneous viruses including avian influenza virus, duck viral enteritis, and goose parvovirus using PCR (data not shown).

### Virus Isolation and Characterization

PCR-positive samples inoculated in SPF-ECE consistently induced stunting, congestion, subcutaneous hemorrhages, and erratic embryonic death after three successive passages. The embryo livers showed either diffused necrotic foci or congestion, and some of the collected allantoic fluid showed greenish-yellow coloration.

**Figure 2:**
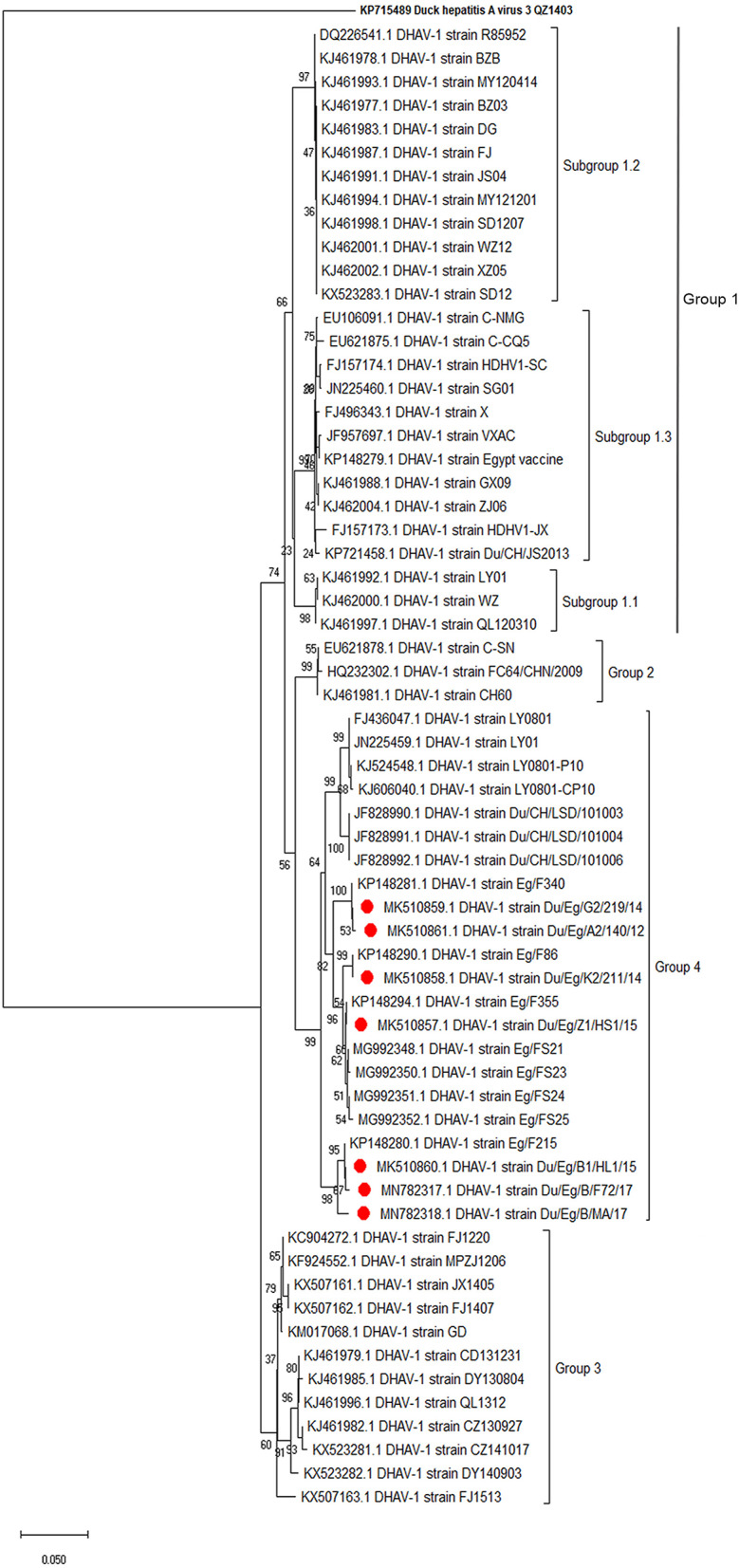
Phylogenetic relatedness of the VP1 gene of duck hepatitis A virus-1 (DHAV-1) isolates to the Egyptian vaccine and other representative Asian strains. Neighbor-joining tree generated with 1,000 bootstraps.

**Figure 3:**
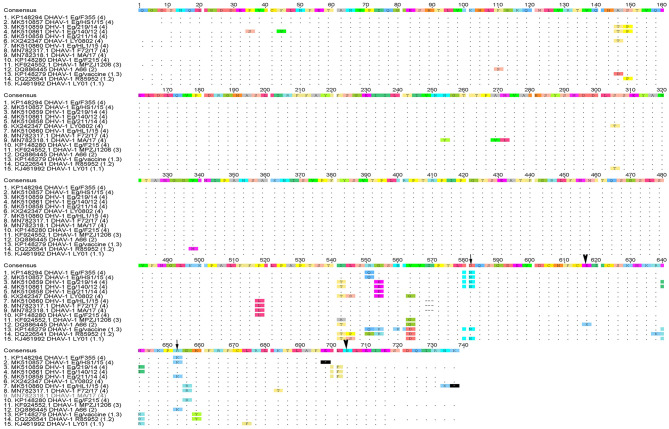
Deduced amino acid sequences of the VP1 gene of duck hepatitis A virus-1 (DHAV-1) isolates of the current study in comparison with selected Egyptian and vaccine strains. Amino acid substitutions D193N, R217K in MK510860 (Eg/HL-1/15) and MK510859 (Eg/F219/14) (arrowheads) and amino acid residues E205, N234 of DHAV-1 virulent strains (arrows) are indicated.

Phylogenetic analyses based on the full-length VP1 gene revealed that DHAV-1 field isolates cluster into one group closely related to circulating Egyptian strains during the same period with accession numbers MK510857-MK510861, MN782317, and MN782318 (Figure 2). The nucleotide identity of obtained isolates and other Egyptian and Chinese strains clustered into genetic group 4 ranged between 94.6 and 96.7%, while amino acid identities were 94.3–97.1%. The nucleotide and amino acid identities to the currently used vaccines were 92.8–93.5% and 94.3–97%, respectively (Table 2). The carboxyl-terminal of VP1 contained two hypervariable regions (HVRs) at 180–194 and 205–219. Amino acid substitutions I180T, G184E, D193N, and M213I were identified in the C-terminus of MK510859 and MK510861 strains. MK510860, MN782317, and MN782318 strains consistently showed deletion mutation at I189 and G218R amino acid substitution. R217K substitution was observed in MK510857 and MK510858 strains. Two other amino acid residues (E205, N234) were observed among virulent strains including those of the current study (Figure 3).

**Table 2:** Nucleotide and amino acid sequence identities of the VP1 gene of duck hepatitis A virus-1 (DHAV-1) isolates from commercial ducklings.

**Strain**		**1**	**2**	**3**	**4**	**5**	**6**	**7**	**8**	**9**	**10**	**11**	**12**	**13**	**14**	**15**	
1.	**MN782317.1 DHAV-1 F72/17 (4)**		98.4	96.0	99.7	95.6	95.8	95.1	96.3	99.3	95.5	93.9	94.4	92.8	92.5	92.4	**Nucleotide identity percentage**
2.	**MN782318.1 DHAV-1 MA/17 (4)**	98.4		95.7	98.6	95.0	95.3	94.6	95.7	98.1	94.6	93.6	93.9	93.1	92.4	92.2	
3.	**MK510857 DHAV-1 Eg/HS1/15 (4)**	96.4	95.8		96.4	97.2	98.6	97.0	100.0	96.8	96.3	93.5	94.4	93.5	92.7	93.0	
4.	**MK510860 DHV-1 Eg/HL-1/15 (4)**	99.6	98.4	97.0		95.3	96.1	95.5	96.7	99.6	95.4	93.6	94.3	93.3	92.4	92.7	
5.	**MK510859 DHV-1 Eg/F219/14 (4)**	94.7	93.9	96.7	94.7		97.2	100.0	97.5	96.2	96.4	93.7	93.8	93.2	93.3	92.8	
6.	**MK510858 DHV-1 Eg/211/14 (4)**	96.6	96.4	98.7	97.1	97.3		97.0	98.8	96.6	96.7	93.4	94.2	93.1	92.6	92.5	
7.	**MK510861 DHV-1 Eg/140/12 (4)**	94.0	93.2	96.1	94.5	100.0	96.6		97.2	95.9	96.0	93.4	93.4	92.7	92.7	92.1	
8.	KP148294 DHAV-1 Eg/F355 (4)	96.4	95.8	100.0	97.0	96.7	98.7	96.1		97.1	96.5	93.8	94.4	93.8	92.9	93.2	
9.	KP148280 DHAV-1 Eg/F215 (4)	98.7	97.4	97.8	99.2	95.9	97.9	95.3	97.8		96.2	94.7	94.7	93.7	93.1	93.1	
10.	KX242347 DHAV-1 LY0802 (4)	95.8	94.3	96.1	95.9	96.9	97.1	96.2	96.1	96.6		93.5	94.4	93.1	93.4	93.4	
11.	KF924552.1 DHAV-1 MPZJ1206 (3)	97.5	96.4	97.8	97.6	96.5	97.9	95.8	97.8	98.3	98.0		96.2	94.4	94.2	94.4	
12.	DQ886445 DHAV-1 A66 (2)	96.0	95.8	97.4	96.5	95.8	97.8	95.2	97.4	96.9	96.9	98.2		95.0	94.9	94.9	
13.	KP148279 DHAV-1 Eg/vaccine (1.3)	94.8	94.3	97.0	95.4	95.5	95.8	94.9	97.0	95.8	95.4	96.2	94.7		96.6	96.6	
14.	DQ226541 DHAV-1 R85952 (1.2)	94.1	93.8	94.8	94.3	95.2	95.0	94.5	94.8	95.0	95.5	95.9	94.7	95.8		96.6	
15.	KJ461992 DHAV-1 LY01 (1.1)	95.3	94.8	96.5	95.8	95.9	95.8	95.3	96.5	96.2	97.5	97.1	96.1	96.2	95.8		
**Amino acid identity percentage**																	

*Isolated strains are indicated (Bold)*.

### Comparative Pathogenicity of Two Duck Hepatitis A Virus-1 Isolates in Pekin and Muscovy Ducklings

#### Clinical Signs and Mortality

**Figure 4:**
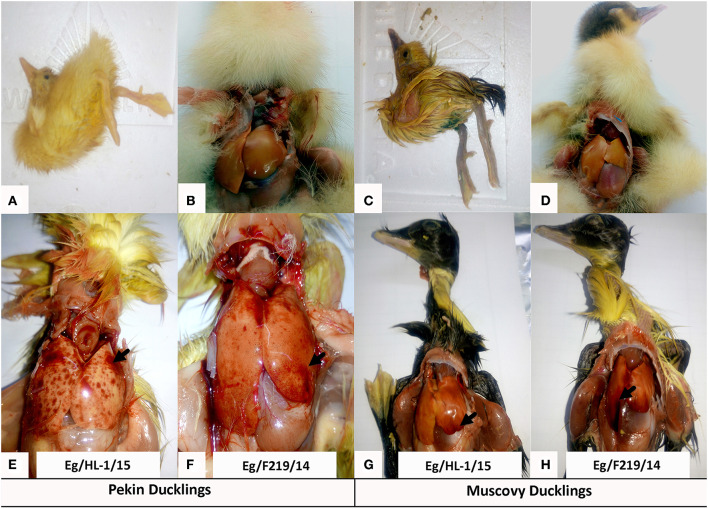
Clinical signs and gross pathological lesions of day-old Pekin and Muscovy ducklings experimentally infected with duck hepatitis A virus-1 (DHAV-1), MK510860 (Eg/HL-1/15), and MK510859 (Eg/F219/14). **(A,C)** Signs of opisthotonos and spasmodic kicking. **(B,D)** Non-infected Pekin and Muscovy controls showed normal liver appearance. **(E,F)** Hemorrhagic spots on the liver surface of Pekin ducklings. **(G,H)** Muscovy livers showed severe congestion.

**Figure 5:**
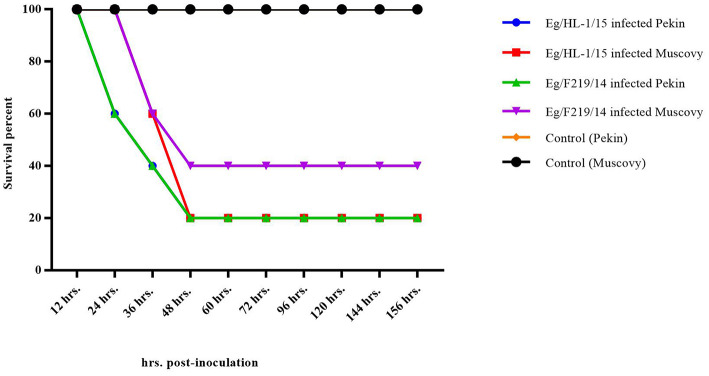
Survival rate of day-old Pekin and Muscovy ducklings experimentally infected with duck hepatitis A virus-1 (DHAV-1), MK510860 (Eg/HL-1/15), and MK510859 (Eg/F219/14).

Both MK510860 (Eg/HL-1/15) and MK510859 (Eg/219/14) isolates induced typical DHAV-1 clinical signs in Pekin and Muscovy ducklings (Figures 4A,C). Mortality rates were 80% in both Pekin challenged groups (Table 3). Pekin ducklings started to die precisely after 24 h post-infection (hpi), and no mortality was recorded after 72 hpi (Figure 5). Livers showed severe petechial hemorrhages on the surface (Figures 4E,F). Similarly, mortality rates of Muscovy ducklings infected with MK510860 and MK510859 strains were 80 and 60%, respectively (Table 3). Muscovy ducklings started to die nearly after 36 hpi, and no mortality was recorded after 96 hpi (Figure 5). Liver gross lesions were limited to congestion, and both DHAV-1 isolates induced milder lesions compared to Pekin ducklings (Figures 4G,H). No gross lesions were recorded in either Pekin or Muscovy control groups (Figures 4B,D).

**Table 3:** Clinical signs, gross lesions, and histopathology scores, and mortality rates in Pekin and Muscovy ducklings experimentally infected with duck hepatitis A virus-1 (DHAV-1) isolates MK510860 (Eg/HL1/15) and MK510859 (Eg/F219/14).

**Observation**	**Challenged**				**Control**	
	**MK510860**		**MK510859**			
	**Pekin**	**Muscovy**	**Pekin**	**Muscovy**	**Pekin**	**Muscovy**
Signs (Opisthotonos/spasmodic kicking)	+++	+++	+++	++	–	–
**Gross Lesions**						
•Liver	+++	++	+++	+	–	–
**Histopathology**						
•Liver	++++	+++	+++	++	–	–
•Pancreas	+	+	+	+	–	–
•Brain	+	+	+	+	–	–
Mortality	8/10 (80%)	8/10 (80%)	8/10 (80%)	6/10 (60%)	0 (0%)	0 (0%)

*Scoring: (–) normal, (+) mild lesion, (++) moderate, (+++) severe, and (++++) very severe*.

#### Histopathological Examination

**Figure 6:**
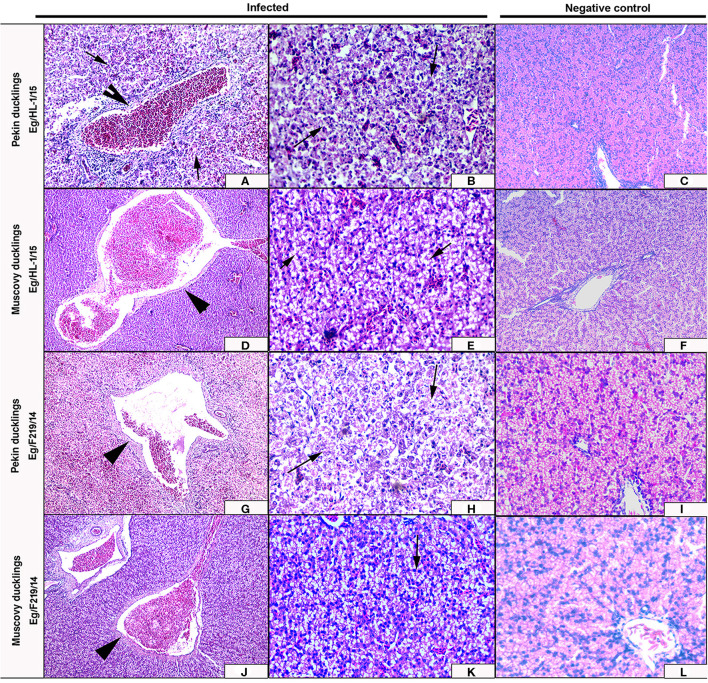
Livers of experimentally infected Pekin and Muscovy ducklings with duck hepatitis A virus-1 (DHAV-1) strains compared to non-infected negative controls. Liver of Pekin ducklings showing congested blood vessels, hepatocellular necrosis (x200) **(A)**, and severe hemorrhage (x200) **(B)**. Liver of Muscovy ducklings showing severely congested blood vessels (x100) **(D)**, and vacuolar degeneration (x400) **(E)**. Liver of Pekin ducklings showing severely congested blood vessels, hemorrhage (x100) **(G)**, and severe necrosis of hepatocytes with few perivascular mononuclear cells infiltration (x400) **(H)**. Liver of Muscovy ducklings showing severely congested blood vessels with proliferation of bile ductules (x100) **(J)**, and vacuolar degeneration (x400) **(K)**. No histological microscopic lesions were observed in the control groups (x200) **(C,F)** and (x400) **(I,L)**.

Livers of Pekin ducklings infected with MK510860 strain showed severe congestion of hepatic blood vessels and hemorrhage (Figure 6A) accompanied by severe degeneration, necrosis, and dissociation of hepatocytes, in addition to multifocal hemorrhage and diffuse infiltration with heterophils and macrophages (Figure 6B). Livers of Muscovy ducklings showed severely congested hepatic blood vessels and vacuolar degeneration (Figures 6D,E).

Livers of Pekin ducklings infected with MK510859 strain showed severe congestion, hemorrhage, and thrombus formation. Perivascular inflammatory cell infiltration was also observed. Necrosis and dissociation of hepatocytes were noticed as well (Figures 6G,H). In Muscovy ducklings, livers showed vacuolar degeneration, congested hepatic blood vessels, and proliferation of bile ductules (Figures 6J,K).

**Figure 7:**
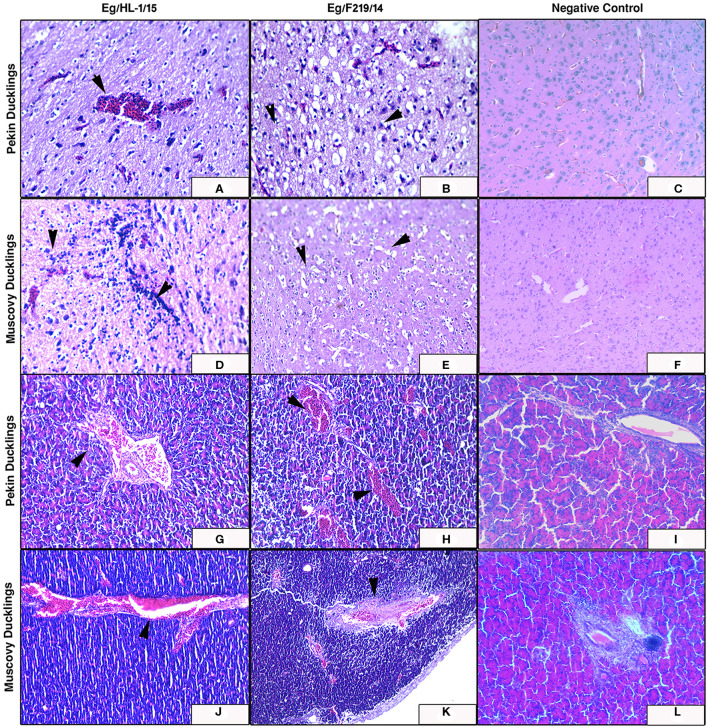
Cerebrum and pancreas of experimentally infected Pekin and Muscovy ducklings with duck hepatitis A virus-1 (DHAV-1) strains compared to non-infected negative controls. Cerebrum of Pekin ducklings infected with Eg/HL-1/15 strain showing cerebral congestion (x200) **(A)**. Cerebrum of Pekin ducklings infected with Eg/F219/14 strain showing neuronal degeneration with perineural edema (x400) **(B)**. Cerebrum of Muscovy ducklings infected with Eg/HL-1/15 strain showing proliferation of glia cells (x400) **(D)**. Cerebrum of Muscovy ducklings infected with Eg/F219/14 strain showing perineural edema (x400) **(E)**. Pancreas of Pekin ducklings infected with Eg/HL-1/15 strain showing congested blood vessels (x200) **(G)**. Pancreas of Pekin ducklings infected with Eg/F219/14 strain showing congested blood vessels (x200) **(H)**. Pancreas of Muscovy ducklings infected with Eg/HL-1/15 strain showing congested blood vessels (x200) **(J)**. Pancreas of Muscovy ducklings infected with Eg/F219/14 strain showing congested blood vessels and thickening of their walls (x200) **(K)**. No histological microscopic lesions were observed neither in the Cerebrums of control groups (x200) **(C,F)** nor in Pancreas (x200) **(I,L)**.

The MK510860 strain induced congestion of blood vessels in both cerebrum and pancreas of experimentally infected Pekin ducklings (Figures 7A,G). In Muscovy ducklings, focal proliferation of glia cells accompanied by perineural and perivascular edema was observed. Pancreas showed mild congestion (Figures 7D,J). Pekin ducklings infected with MK510859 strain showed congestion of pancreatic blood vessels and vacuolar degeneration of few acini. Congestion of cerebral blood vessels, perineural edema, and focal demyelination were observed. (Figures 7B,H). The cerebrum of infected Muscovy ducklings exhibited perivascular edema, while the pancreas showed wall thickening and congestion of blood vessels (Figures 7E,K). No histopathological microscopic lesions were observed in either Pekin or Muscovy control groups (Figures 6, 7).

## Discussion

DHAV-1 disease continues to be a threat to duck farms because of the high mortality associated with the disease which often exceeds 50% and can reach 95% under field conditions (18, 29). In this study, we investigated DHAV in suspected duck farms and backyards in five provinces in Egypt. The majority of the investigated field outbreaks were in ducklings aged below 1 week (22 out of 30). The severity of DHAV infection in ducks is known to be an age-dependent infection where the highest mortalities are reported in young ducklings due to their immature immune systems (30). The expression of the immune-related genes in day-old ducklings is insufficient to protect them from virus replication (31). Seven samples confirmed for DHAV-1 by 5′UTR and VP1-specific RT-PCRs were isolated on 9-day-old SPF eggs. We were not able to confirm the two samples originated from Muscovy ducklings by VP1-specific RT-PCR (one was negative for 5′UTR, and the other one was positive for 5′UTR and negative for VP1 of DHAV-1); therefore, the seven isolated samples were originating from Pekin (*n* = 6) and Mulard (*n* = 1).

The VP1 is the most external and immunodominant capsid protein that interacts with cellular receptors to elicit neutralizing antibodies (32). Additionally, VP1 sequence analysis is the currently used tool to characterize DHAV on a molecular basis. Hence, the conducted VP1-based phylogenetic analysis revealed that DHAV-1 strains are classified into four main groups (17). The current study isolates clustered with the previously reported Egyptian and Chinese virulent viruses into genetic group 4 (16). Subgrouping of Egyptian DHAV-1 strains into A, B1, B2, and C groups according to their geographic distribution was reported (18); however, we could not define a specific geographic correlation of the isolated DHAV-1 strains. This is probably due to the uncontrolled bird movement across all the Egyptian provinces. The isolated viruses were genetically distant from genetic group 1 comprised of a variety of DHAV-1 classic and vaccine strains (e.g., R85952, acc.no. DQ226541). Also, the Egyptian DHAV-1 viruses are distant from genetic group 2 (attenuated Chinese isolates and recent commercial vaccinal strains, e.g., A66 acc.no. DQ886445) and genetic group 3 [DHAV-1a variant strains MPZJ1206 (KF924552), FJ1220 (KC904272), and JX1405 (KX50716) isolated from Muscovy ducklings, pigeon, and geese, respectively].

In-depth genetic analysis revealed that the carboxyl-terminal of VP1 contained two hypervariable regions (HVRs) at 180–194 and 205–219 among the different DHAV-1 strains. Some of these differences were previously linked to the changes of the virulence criteria of DHAV-1 (25, 32); however, the virulence of DHAV-1 virus was not only attributed to the C-terminus of VP1 (33). Conversely, Chinese virulent and attenuated DHAV-1 strains with identical VP1 showed minor differences in the VP0 and VP3 (34), suggesting that DHAV-1 virulence may not involve the capsid polypeptide and other genomic regions, besides the VP1 gene, are responsible for the DHAV-1 virulence.

DHAV-1 infections are associated with acute disease in Pekin, Mallard, and some hybrid breeds only, however, Muscovy ducklings were considered as healthy carriers. Interestingly, recent variant strains belonging to the genetic group 3 were associated with pancreatitis and encephalitis, accompanied by 25–40% mortalities with no significant liver lesions (20, 21, 24). The pancreatitis-type DHAV-1 in Muscovy ducklings is now defined to be a distinct type from conventional hepatitis type (22, 23, 35). Other variant viruses also caused hepatitis in pigeons and egg drop syndrome in laying ducks (36, 37). The diversity of the translation systems utilized by chicken and duck embryos was suggested to play an important role in the differences between virulent and attenuated DHAV-1 strains replication efficiency in the infected host tissues (30). Additionally, Muscovy ducks (*Cairina moschata domestica*) were found to be genetically different and constitute a distinct genetic group from other duck breeds (*Anas platyrhynchos domesticus*) using Random Amplified Polymorphic DNA Analysis (38). However, the role of such breeds' genetic diversity in the susceptibility to DHAV-1 infection remains unclear.

In Egypt, though DHAV-1 was detected in clinical samples collected from Muscovy ducklings with mortality rates of 15–50%, all the isolated strains belonged to the genetic group 4 (i.e., not variant). The pathogenicity of the virus isolates was not evaluated (16, 18). Therefore, in the current study, we conducted an experimental pathogenicity model in day-old Pekin and Muscovy ducklings using two DHAV-1 strains belonging to the genetic group 4 (MK510859 and MK510860). Unexpectedly, both duck breeds exhibited characteristic DHAV-1 clinical signs of lethargy, ataxia, and opisthotonos. No significant differences were detected in either mortality rates or gross pathological pictures of the two strains in each species. Consistently, the mortality rates were higher, and the deaths started as early as 24 h post-infection in Pekin compared to Muscovy ducklings.

The DHAV-1 strains induced severe hepatic petechial hemorrhages in experimentally infected Pekin compared to liver congestion in Muscovy ducklings. The histopathological findings supported the gross pathological observations, where livers of experimentally infected Muscovy ducklings exhibited severely congested blood vessels accompanied by vacuolar degeneration. In contrast, Pekin ducklings showed typical severe degeneration and necrosis with dissociation of hepatocytes, multifocal hemorrhages, and severe congestion of hepatic blood vessels (19). Both breeds showed similar brain and pancreatic histopathology including focal proliferation of cerebrum glia cells, marked perineural and perivascular edema, focal demyelination, in addition to signs of pancreatic congestion. The observed hepatic hemorrhage was previously reported in DHAV-1 (genetic group 1) experimentally infected Muscovy ducklings (24). However, hepatic microscopical lesions were more severe (degeneration and necrosis of hepatocytes, substantial infiltration of inflammatory and red blood cells) compared to the current study. The findings of pathogenicity study in Muscovy ducklings are consistent with previous studies declaring recent alteration of pathogenicity criteria of DHAV-1 (31, 35, 39, 40). Differences in the severity of gross and microscopic lesions between duckling species could be attributed to the degree of host adaptability considering that both tested DHAV-1 strains were isolated from Pekin ducklings.

Taken together, these findings highlighted the possibility of infection of Muscovy ducklings with DHAV-1. To the best of our knowledge, this is the first report of comparative pathogenicity of DHAV-1 isolates in Pekin and Muscovy ducklings in Egypt and the Middle East region. The genetic divergence between recent Egyptian strains and commercial vaccines urges conducting vaccine efficacy evaluation and/or new vaccine candidates' development. Finally, studying the relationship between the susceptibility to DHAV-1 infection and duck breeds biodiversity can contribute to a better understanding of pathogenicity variation of different DHAV-1 groups.

## Data Availability Statement

The datasets for this study can be found in GenBank, under accession numbers https://www.ncbi.nlm.nih.gov/nuccore/MK510857; https://www.ncbi.nlm.nih.gov/nuccore/MK510858; https://www.ncbi.nlm.nih.gov/nuccore/MK510859; https://www.ncbi.nlm.nih.gov/nuccore/MK510860; https://www.ncbi.nlm.nih.gov/nuccore/MK510861; https://www.ncbi.nlm.nih.gov/nuccore/MN782317; https://www.ncbi.nlm.nih.gov/nuccore/MN782318.

## Ethics Statement

All experimental procedures were reviewed and approved by the Animal Care and Use Committee of the Faculty of Veterinary Medicine, Damanhour University, Egypt (Approval no. DMU/VetMed-2018/0213).

## Author Contributions

HE, AE, AS, WK, and AA: conceptualization. IH, AA, MA, and WK: methodology. IH, HE, AE, AS, AA, WK, and MZ: investigation. IH, AA, AS, and HE: data analysis. All authors: resources and writing and editing. IH, HE, AE, AS, and AA: data curation. IH, MA, and AA: histopathology. IH, AA, WK, and HE: writing—original draft preparation.

## Conflict of Interest

The authors declare that the research was conducted in the absence of any commercial or financial relationships that could be construed as a potential conflict of interest.
