# Supplementary material for: Comparative Pathogenicity of Duck Hepatitis A Virus-1 Isolates in Experimentally Infected Pekin and Muscovy Ducklings
Source: Front Vet Sci. 2020 May 15;7:234. doi: 10.3389/fvets.2020.00234 (PMC7326108; doi:10.3389/fvets.2020.00234)
Supplement: Supplementary file 1 [file Table_1.DOCX]

**Supplementary table 1. Clinical history of collected samples**

| **Sample Code** | **Year** | **Location** | **Breed** | **Age (day)** | **Flock Size** | **Mortality (%)** | **5' UTR specific RT-PCR** | **VP1 specific RT-PCR** |
| --- | --- | --- | --- | --- | --- | --- | --- | --- |
| F72/17 | 2017 | Beheira | Pekin | 5 | 2000 | 70 | + | + |
| MA/17 | 2017 | Beheira | Pekin | 5 | 900 | 30 | + | + |
| F70/17 | 2017 | Alexandria | Pekin | 6 | 1500 | 22 | - | na |
| F2/17 | 2017 | Kafr El Sheikh | Pekin | 3 | 1000 | 32 | - | na |
| F220/16 | 2016 | Alexandria | Pekin | 7 | 2000 | 75 | - | na |
| F100/16 | 2016 | Gharbia | Pekin | 15 | 2000 | 10 | - | na |
| F55/16 | 2016 | Gharbia | Pekin | 7 | 1000 | 70 | - | na |
| F296/15 | 2015 | Beheira | Muscovy | 6 | 5000 | 30 | - | na |
| HS-1/15 | 2015 | Giza | Pekin | 3 | 3000 | 40 | + | + |
| HS-2/15 | 2015 | Giza | Pekin | 5 | 2000 | 26 | - | na |
| HL-1/15 | 2015 | Beheira | Pekin | 3 | 1000 | 14 | + | + |
| F209/15 | 2015 | Gharbia | Pekin | 5 | 500 | 40 | - | na |
| F287/14 | 2014 | Alexandria | Muscovy | 6 | 2500 | 10 | + | - |
| F283/14 | 2014 | Kafr El Sheikh | Mulard | 11 | 1500 | 33 | + | - |
| F229/14 | 2014 | Gharbia | Pekin | 7 | 2950 | 53 | - | na |
| F219/14 | 2014 | Gharbia | Pekin | 6 | 2000 | 30 | + | + |
| F211/14 | 2014 | Kafr El Sheikh | Mulard | 8 | 1500 | 20 | + | + |
| F66/14 | 2014 | Alexandria | Pekin | 13 | 800 | 21 | - | na |
| AB-1/14 | 2014 | Beheira | Pekin | 5 | 1000 | 80 | + | - |
| AB-2/14 | 2014 | Beheira | Mulard | 12 | 500 | 50 | - | na |
| HL-2/14 | 2014 | Beheira | Pekin | 7 | 1200 | 18 | - | na |
| 29M/14 | 2014 | Kafr El Sheikh | Mulard | 3 | 3000 | 40 | - | na |
| F29/13 | 2013 | Kafr El Sheikh | Pekin | 8 | 2000 | 45 | - | na |
| F50/13 | 2013 | Gharbia | Pekin | 21 | 3000 | 55 | - | na |
| F58/13 | 2013 | Gharbia | Pekin | 7 | 1500 | 23 | - | na |
| F60/13 | 2013 | Beheira | Mulard | 7 | 1000 | 19 | - | na |
| F140/12 | 2012 | Alexandria | Pekin | 7 | 2000 | 14 | + | + |
| F122/12 | 2012 | Beheira | Pekin | 3 | 1000 | 5 | - | na |
| F111/12 | 2012 | Giza | Pekin | 9 | 3850 | 25 | - | na |
| F79/12 | 2012 | Alexandria | Pekin | 3 | 4000 | 30 | - | na |
